# Historical Statement of the Galvanic Discovery, and of the Publications That Have Appeared on That Subject

**Published:** 1802-01

**Authors:** 


### Historical Statement of the Galvanic Discovery, and of the Publications that have appeared on that Subject.

THE difcovery of Mr. Galvani is undoubtedly one of the moft important that has ever been made in the ample field of natural philofophy, and from the extenfive views which it opens to the enquiries of naturalifts, it feems to deferve their utmoft attention. Although we may fuppofe the principal works on that interefting fubject to be known to profeffional naturalifts, yet it may not be improper to give an account of all the tranfactions on that moft important phenomenon to the public at large, which, however, we purpofe to be merely hiftorical, becaufe it would be unjuft to criticife the more early theories and hypothefes relating to that object, guided by the late difcoveries with which it has fince been enlightened.

The firft traces towards the difcovery is found in the following book, where, however, it was overlooked, and foon buried in oblivion.

Sulzer's Theorie der angenehmen und unangenehmen Empfindungen ; *i. e.* Theory of agreeable and difagreeable Senfations, tranflated from the French into German, under the direction of the author, with additional remarks in Sammlung Vermifchter Schriften zur Beforderung der Schonen Wiffenfchaften ; *i. e.* Collection of Mifcellaneous Writing for the Improvement of Belles Lettres and Free Arts, Vol 5, No. I, Berlin, 1762 ; and alfo in J. G. Sulzer's Vermifchte Schriften, *i. e.* Mifcellaneous Writings. Leipzic, 1773.—"When two pieces of metal," fays Mr. Sulzer, "one of lead and the other of filver, are joined together fo that their edges make one furface, a certain fenfation will be produced on applying it to the tongue, which comes near to the tafte of martial vitriol, whereas each piece by itfelf betrays not the leaft traces of that tafte. It is not improbable, he continues, but that by the combination of the two metals a folution of either of them may have been produced, in confequence of which the diffolved particles penetrate into the tongue ; or, we may conjecture, that the combination of thefe metals occafions a trembling motion in their refpective particles, which, exciting the nerves of the tongue, caufes that particular fenfation."—This hint, however, feems to have been difregarded till Galvani publifhed the following work :

Aloyfii Galvani de Viribus Electricitatis in motu Mufculari Commentarius, 1791, pp. 58, 4to. Bologna, for the Inftitute of Sciences ; which was foon followed by other publications relating to animal electricity, viz.

Lettera

Lettera del Dottore Eufebio Valli ful l'Elettricità Animale ad un fuo Amico. Pavia, 1792, pp. 15, 4to.

Memoria ful l'Ectricità Animale, inferité nel Giornale Fifico-Medico del Sigr. Brugnatelli; *i. e.* Memoir on Animal Electricity, inferted in the Phyfical and Medical Journal of M. Brugnatelli. Pavia, 1792, pp. 147, 8vo.

A. Galvani Abhandlung ueber die Kraefte der Thierifchen Electricitat auf die Bewegung der Mufkeln, &c. *i. e.* Treatife on the Effects of Animal Electricity on Mufcles, together with fome writings of Meffrs. Valli, Carminati, and Volta, on the fame fubject; a tranflation, edited by Dr. J. Meyr, with four plates, 1793, pp. 183. Prague, for Calve, 8vo.

Schriften ueber die Thierifche Electricitæt; *i. e.* Memoirs on Animal Electricity, by D. Alexander Volta; tranflated from the Italian by Dr. J. Meyr, 1793, pp. 144. Prague, for Calve, 8vo.

The work of the celebrated Galvani is divided into four parts, the firft of which treats of the effect of electricity, which is produced by art; the fecond of the action of atmofpherical electricity; the third of the effect of what he calls animal electricity; and the fourth contains fome conjectures and conclufions. But he confeffes, with an ingenuity which always attends true merit, how much of his difcovery is owing to accident, and he never diffembles the falfe conclufions to which he was mifled by the firft view of each new phenomenon.

Whilft Mr. Galvani was diffecting a frog on the table, whereon accidentally ftood an electrical machine, one of his pupils happened to touch the nervus cruralis of the frog with the point of the diffecting knife, upon which, immediately, the mufcles of all the members were convulfively contracted. Another ftanding by, thought he obferved that this phenomenon took place when a fpark was drawn from the conductor of the machine, an idea which was afterwards confirmed; for, on touching the fame nerve of another frog, and likewife pricking it in order to affure himfelf whether it was owing to his having accidentally wounded the nerve, without drawing a fpark at the fame time, not any motion enfued; but if the nerve was touched with the point of a knife, at the time when he had ordered a fpark to be taken from the machine, the fame phenomenon appeared again. On repeating the experiment with the fame knife, the motions were fometimes ftronger, fometimes weaker, and fometimes difappeared entirely, which was found to arife from the manner in which he happened to hold the knife; becaufe when he held the handle, which was of bone, the animal remained motionlefs; but as foon as he touched the metallic part the contractions were immediately produced. In order to determine determine whether this phenomenon depended on the idioelectric nature of the dry bone, or on the conducting property of metal, Mr. Galvani changed the knife for a clean glafs tube, and for an iron cylinder, but the above phenomenon never appeared on applying the glafs tube to the nerve, even when very ftrong fparks were drawn, whereas it was immediately produced by the leaft fpark on applying the iron cylinder. Thus Mr. Galvani found it confirmed, that for producing the above phenomenon the contact of a conducting body with the nerve was requifite.

When the iron cylinder was applied to the nerve, without being held by the hand of any body, the drawn fpark occafioned no motion, whereas the contraction came on when, inftead of the cylinder, he took a long iron wire, fo that a certain length and extenfion of the conducting body feemed to be required for effecting the above phenomenon. These conducting bodies were called by Mr. Galvani nervous conductors. The experiment likewife fucceeded at a diftance, by very long infulated conductors in animals prepared for that purpofe, particularly when a conducting tube was hung at the feet of the frog, communicating with the floor; thefe conductors he diftinguifhed by the name of mufcular conductors. After having made a great number of experiments in a different manner, viz. by interrupting the free courfe of electricity, by coating the nervous conductor to their ends with an electric fubftance, and by applying negative electricity, the electrophor, &c. the effect of atmofpherical electricity on mufcular motion remained to be examined. To this end he raifed a long and proper conductor, which was infulated on the roof of a houfe, from which frogs, or the legs of warm blooded animals were hung by the nerves; another conductor being attached to their feet went into the water of a well. As foon as it began to lighten, the mufcles were feized with violent and repeated contractions, which, like the lightning, preceded the thunder; thefe contractions even enfued, when no mufcular conductor had been applied, and the nervous conductor not infulated; they where even obferved when the conductor was raifed on lower places, and in gloomy weather. The experiments fucceeded with dead animals as well as with living, but the mere lightning without thunder produced no movements.

Thefe curious experiments happened to give rife to the proper Galvanic difcovery. Mr. Galvani being curious to know what effect atmofpherical electricity might have in quiet and clear weather, he fufpended fome frogs on metal hooks, fixed in the fpine of the back, from the iron rails of his garden, and he obferved thofe contractions, not only when it lightned, but alfo alſo in clear and quiet weather. At firſt he thought, that the cauſe of theſe contractions might ariſe from changes in the electricity of the atmoſphere, but upon a more minute examination, he found that on bending the hook with which the ſpinal marrow was perforated, towards the iron rails, in order to ſee whether muſcular motion might be thus produced, and whether any difference or change in the ſtate of atmoſpherical electricity would manifeſt itſelf, he began to conceive, that the contractions did not relate to this ſtate of the atmoſphere. Having however, only ſeen theſe contractions take place in open air, he was, notwithſtanding, inclined to aſcribe them to the atmoſpherical electricity, which running over into the animal, may there be accumulated; and he imagined, that hence it might be vehemently diſcharged at the contact of the hook with the iron rail; but he was ſoon undeceived. Having placed a frog upon an iron plate in his room, he happened to preſs it againſt the plate with his diſſecting forceps, upon which the contractions immediately took place. The experiment ſucceeded with all metals, but never on employing non-conducting ſubſtances. Induced by theſe experiments, our author began to ſuſpect the animal to poſſeſs an electricity of its own; a conjecture which appeared to him to be confirmed by the phenomena of a circulation from the nerves to the muſcles, ſimilar to that taking place in the Leyden phial; for on holding a frog that had been previouſly prepared for the experiment by a hook fixed in the ſpine of the back, with one hand, ſo as to let the feet reach a ſmall ſilver cup, and on touching the cup with the other hand by means of a metallic body, the animal fell into violent convulſions. If one perſon held the prepared frog while another touched the cup, no movements were excited, but they immediately enſued when they took hold of each other. If both laid hold of a glaſs tube, the contractions were likewiſe not excited, but they came on when they took an iron cylinder inſtead of the glaſs tube. The inveſtigation of this ſubject was farther proſecuted by a ſeries of experiments. He firſt placed a frog on an electric plate, and touched the animal ſometimes with a conducting arch of iron, ſometimes with an arch but for a part electrical, ſo that he brought one arm in contact with the copper hook which ſecured the animal, the other arm to the femoral muſcles; the movements were produced in the firſt experiment, but in the other, the animal remained motionleſs. Hence he concluded, that the motions proceeding on the iron plate had been owing to a ſimilar arch repreſented by that plate.

